# Metabolomic profiling to dissect the role of visceral fat in cardiometabolic health

**DOI:** 10.1002/oby.21488

**Published:** 2016-04-30

**Authors:** Cristina Menni, Marie Migaud, Craig A. Glastonbury, Michelle Beaumont, Aikaterini Nikolaou, Kerrin S. Small, Mary Julia Brosnan, Robert P. Mohney, Tim D. Spector, Ana M. Valdes

**Affiliations:** ^1^ Department of Twin Research and Genetic EpidemiologyKings College LondonLondonUK; ^2^ School of PharmacyQueen's University BelfastBelfastUK; ^3^ Pfizer Worldwide Research and Development, Clinical Research StatisticsGroton, ConnecticutUSA; ^4^ Metabolon, Inc.DurhamNorth CarolinaUSA; ^5^ Academic Rheumatology Clinical Sciences Building, Nottingham City HospitalNottinghamUK

## Abstract

**Objective:**

Abdominal obesity is associated with increased risk of type 2 diabetes (T2D) and cardiovascular disease. The aim of this study was to assess whether metabolomic markers of T2D and blood pressure (BP) act on these traits via visceral fat (VF) mass.

**Methods:**

Metabolomic profiling of 280 fasting plasma metabolites was conducted on 2,401 women from TwinsUK. The overlap was assessed between published metabolites associated with T2D, insulin resistance, or BP and those that were identified to be associated with VF (after adjustment for covariates) measured by dual‐energy X‐ray absorptiometry.

**Results:**

In addition to glucose, six metabolites were strongly associated with both VF mass and T2D: lactate and branched‐chain amino acids, all of them related to metabolism and the tricarboxylic acid cycle; on average, 38.5% of their association with insulin resistance was mediated by their association with VF mass. Five metabolites were associated with BP and VF mass including the inflammation‐associated peptide HWESASXX, the steroid hormone androstenedione, lactate, and palmitate. On average, 29% of their effect on BP was mediated by their association with VF mass.

**Conclusions:**

Little overlap was found between the metabolites associated with BP and those associated with insulin resistance via VF mass.

## Introduction

Visceral obesity has been linked to insulin resistance, dyslipidemia, and increased cardiovascular risk 1. Adipocytes in visceral fat (VF) secrete numerous adipokines responsible for regulating different physiological systems involved in energy homeostasis and endocrine and immune function 2, which are likely to determine the link between higher VF mass and reduced cardiometabolic health or increased risk of metabolic syndrome.

A number of metabolomic studies have examined associations between metabolites and type 2 diabetes (T2D) 3, 4, 5, 6, 7, 8 and between metabolites and blood pressure (BP) regulation 9. Several amino acids (isoleucine, leucine, valine, tyrosine, and phenylalanine) plus other metabolites have been consistently associated with diabetes 3, 4, 5, 6, 7, 8. Metabolomic profiling of BP has also been carried out and has found that a dicarboxylic fatty acid is functionally involved in increased BP 9. Both increased BP and T2D are part of the metabolic syndrome, and increased VF mass is a risk factor for both.

Excess VF is strongly associated with impaired suppression of free fatty acid (FFA) release in response to insulin 2, as well as with hypertriglyceridemia and low concentrations of high‐density lipoprotein cholesterol. The high FFA concentrations in adipocytes and the adipose tissue‐resident macrophages result in more proinflammatory cytokines, but less adiponectin. These cytokine changes induce insulin resistance in muscle and liver and play a major role in the pathogenesis of endothelial dysfunction, a mechanism involved in increased BP 10. Increased VF may also selectively affect hepatic metabolism via greater portal vein FFA delivery 11. High levels of FFA may also directly affect mitochondrial function and energy metabolism, which are known to be impaired in T2D: when a long‐chain fatty acid enters a cell, a CoA group is added to the fatty acid by fatty acyl‐CoA synthase, forming long‐chain acyl‐CoA. The fatty acid is then transported across the inner mitochondrial membrane. The long‐chain acyl‐CoA enters the fatty acid β‐oxidation pathway, which results in the production of one acetyl‐CoA from each cycle of fatty acid β‐oxidation. This acetyl‐CoA then enters the mitochondrial tricarboxylic acid (TCA) cycle 12. This overall metabolic process is known to be dysfunctional in individuals with T2D 13.

Metabolomic profiling of VF mass may help identify some of the pathways underlying the metabolite associations found with T2D and hypertension (HTN). We hypothesized that some of the metabolites so far linked to T2D and BP by metabolomic profiling 3, 4, 5, 6, 7, 8, 9 may be acting via VF mass and that they may reveal some of the molecular pathways that are altered in individuals with high VF mass that give rise to metabolic dysfunction resulting in T2D and high BP.

## Methods

We analyzed data from 2,401 female twins from the TwinsUK cohort for whom nontargeted metabolomics analysis was available along with glucose/diabetic information, as well as VF measurement via dual‐energy X‐ray absorptiometry (DXA) scans 14. All individuals were used for all metabolomic versus VF analyses regardless of their fasting glucose levels. The St. Thomas' Research Ethics Committee approved the study (EC96/439 TwinsUK), and all participants provided informed written consent.

### VF measurement by DXA

Measurements of whole body composition were performed for 3,457 female twins aged 40 to 80 years using the DXA fan‐beam technology (Hologic QDR; Hologic, Inc., Waltham, MA). Subjects were positioned in a standardized manner, in a supine position with the clothes removed and wearing a gown. The DXA machine was calibrated on a daily basis using a spine phantom and on a weekly basis using a step phantom, as suggested by the manufacturer. The scans were analyzed using the QDR System Software Version 12.6.

Regions‐of‐interest were defined manually by the same operator following the Standard operating procedure (SOP), which was derived from the manufacturer's guidelines. The lower horizontal margins were placed above the pelvis, just above the iliac crest, while the upper horizontal margins were placed at the half of the distance between the acromions and the iliac crest. The vertical margins were adjusted just at the external borders of the body so that all the soft tissue was included.

This DXA‐based measurement of VF has been validated against VF measured by CT scan 15 and shown to be reliable and reproducible. In a subset of 63 individuals from the TwinsUK cohort, we find that the correlation between VF measured by DXA and by CT scan is of 83% in line with published data.

#### Homeostasis model assessment‐estimated insulin resistance (*HOMA‐IR)*

Fasting insulin and glucose levels were measured for the twin cohort using the same methods as previously described 16. HOMA‐IR was calculated by multiplying overnight fasting plasma insulin (FPI) with overnight fasting plasma glucose (FPG), then dividing by the constant 22.5, i.e. HOMA‐IR = (FPI × FPG)/22.5 17.

### HTN and T2D definitions

T2D cases were defined as individuals with fasting glucose levels ≥7 mmol/L at time of initial sampling and at subsequent visits, while T2D “super controls” were defined as subjects with fasting glucose levels between 3.9 mmol/L and 5 mmol/L, as was done for the original metabolomic study of T2D 5. HTN was defined using the standard stage 1 cut‐off systolic BP (SBP) ≥140 mm Hg or diastolic BP (DBP) ≥90 mm Hg.

### Metabolomics measurements

Nontargeted metabolite detection and quantification was conducted by the metabolomics provider Metabolon, Inc. (Durham) on 2,401 fasting plasma samples from participants in the TwinsUK study, as described previously 18. The metabolomic dataset measured by Metabolon includes 280 known metabolites containing the following broad categories—amino acids, acylcarnitines, sphingomyelins, glycerophospholipids, carbohydrates, vitamins, lipids, nucleotides, peptides, xenobiotics, and steroids.

### Muther expression data

The Muther gene expression dataset included in this study consists of 772 abdominal fat samples analyzed with the Illumina Human HT‐12 V3, as previously described 19.

### Statistical analysis

Statistical analysis was carried out using Stata version 11. We inverse‐normalized the metabolomics data and excluded metabolic traits with >20% missing values. Metabolite associations with VF were assessed by random intercept linear regressions adjusted for age, BMI, height 2, metabolite batch, DXA batch, and family relatedness. We adjusted for multiple testing using Bonferroni correction resulting in a significant threshold of 1 × 10^−4^.

We first tested the known metabolites we previously associated with T2D 5 for correlation with VF. As VF is not only a risk factor for T2D but has been also implicated in cardiovascular disease risk, we used a stepwise backward regression model to identify a set of metabolites that were significantly associated with VF using *P* < 0.01 as cut‐off threshold.

Association of selected metabolites with gene‐expression levels in fat were tested using random intercept linear regression adjusting for age, BMI, metabolite batch, expression batch, and family relatedness.

### Metabolite association mediated by VF

The proportion of the variance of the clinically relevant trait (HOMA‐IR or SBP) was first calculated explained for each metabolite after taking into account all covariates (age, sex, BMI, height, family relatedness, and metabolite batch). We call this quantity $r_{x}^{2}$. The proportion of the variance for the same trait explained by the metabolite was then after taking into account the same covariates as above but adjusting also for VF mass $r_{y}^{2}$. The percentage of the metabolite association mediated by VF ( $r_{y}^{2}$) was calculated as the proportion of the variance of the clinical trait that is due to the metabolite's association with VF, namely 1 ‐ ( $r_{x}^{2}$/ $r_{y}^{2}$).

## Results

The descriptive characteristics of the study participants are presented in Table 1. The DXA measure of VF mass is strongly associated with age, HOMA‐IR, T2D, BP, and BMI (Table 1). The strongest association is seen with BMI which is correlated with VF mass with an *r* ^2^ = 0.617. The unadjusted association with T2D per standard deviation (SD) of VF mass (normalized) is OR_T2D_ [95% CI] = 3.01 [2.35‐3.86]; *P* < 3.6 × 10^−18^. The association remains significant after adjustment for BMI and age (OR_T2D_ = 2.02 [1.36‐3.00]; *P* < 5.2 × 10^−4^). VF mass is also correlated with both systolic and diastolic BP and remains associated with risk of HTN after adjustment for age and BMI (OR_HTN_ = 1.16 [1.02‐1.30] *P* < 0.015).

**Table 1:** Demographic characteristics of the study population

	Mean (SD)	VF Q1	VF Q2	VF Q3	VF Q4	Associated with VF mass, *P* value^a^	
**Visceral fat mass (g)**	568. 2 (298.8)	1.28‐332.7	332.8‐523.0	532.1‐753.4	753.5‐1,725		
**Age (yr)**	56.91 (11.57)	50.86 (12.75)	57.96 (11.33)	58.84 (10.40)	59.94 (9.313)	5 × 10^−59^	b
**BMI (kg/m^2^)**	26.30 (4.90)	21.8 (2.20)	24.4 (2.41)	27.0 (3.08)	31.7 (4.77)	1 × 10^−300^	c
**Fasting HOMA‐IR**	0.98 (0.71)	0.56 (0.31)	0.73 (0.48)	0.99 (0.60)	1.50 (0.86)	3 × 10^−28^	d
**T2D cases: super controls** e **(%)**	93:1,145 (8.1%)	8:297 (3%)	18:277 (6%)	9:254 (3%)	58:217 (21%)	0.000519	d
**Systolic blood pressure (mm Hg)**	127.4 (16.4)	119.4 (14.4)	126.4 (15.6)	130.2 (16.2)	133.5 16	2.14 × 10^−5^	d
**Diastolic blood pressure (mm Hg)**	77.9 (9.6)	74.1 (9.1)	76.9 (9.1)	79.1 (9.3)	81.5 (9.6)	1.97 × 10^−9^	d
**Hypertension**	21.9%	13.4%	18.5%	21.8%	33.7%	0.014	d
***n***	2,401	600	601	600	600		
**Female**	100%	100%	100%	100%	100%		

Data are presented as mean (SD) unless otherwise specified.

a *P* value for association between inverse normalized VF mass and continuous traits by linear or logistic regression adjusted for covariates.

b Adjusted for BMI.

c Adjusted for age.

d Adjusted for age and BMI.

e Super controls are individuals with fasting glucose <5 mmol/L.

VF Q, quartiles of the visceral fat mass distribution in 2,401 individuals from the TwinsUK cohort; HOMA‐IR, homeostasis model assessment‐estimated insulin resistance T2D, type 2 diabetes.

**Figure 1:**
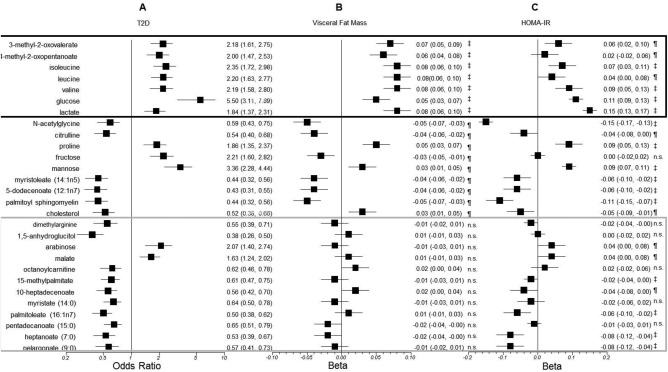
Association of selected metabolites with (**A**) T2D, (**B**) visceral fat mass, and (**C**) homeostasis model assessment‐estimated insulin resistance (HOMA‐IR). The metabolites are those previously reported to be associated with T2D with *P* < 0.0001. The odds ratio with T2D and the coefficients *β* and the 95% confidence intervals of the linear regression with visceral fat mass and log HOMA‐IR adjusted for covariates are shown for each metabolite. *P* values are ‡*P* < 0.0001, ¶0.0001 *< P* < 0.05, and n.s. *P* > 0.05.

We first investigated which metabolites are correlated with both VF mass and T2D. Of the 28 known metabolites we previously identified to be associated with T2D (5) (Figure 1 and Supporting Information Table S1), seven of them including glucose and three branched‐chain amino acids (BCAAs) are also associated with VF mass (after adjusting for multiple testing). At a nominal significance level (*P* < 0.05), 16 of the 28 metabolites are associated with both VF and T2D, and all of them except fructose and 4‐methyl 2‐oxopentaoate are also associated with HOMA‐IR (Figure 1 and Supporting Information Table S1). Twelve metabolites associated with T2D, however, show no correlation with VF mass (see Figure 1).

### Metabolites associated with both T2D and VF mass

Glucose, lactate, valine, leucine, isoleucine, 3‐methyl‐2‐oxovalerate, and 4‐methyl‐2‐oxopentanoate are all associated with T2D and VF. All these metabolites are associated with VF mass independently of T2D status (Figure 1 and Table 2) and all these metabolites, except glucose (which was not tested, being part of the definition of HOMA‐IR), remain significantly associated with VF mass after adjusting for HOMA‐IR and after adjustment for total fat mass. We assessed whether the association between these metabolites and insulin resistance was mediated by VF mass. On average, 38.5% of the variance of HOMA‐IR explained by the metabolites is accounted for by the association with VF mass (Table 2). This value, however, ranges from 19.4% for lactate to 61.3% for 4‐methyl‐2‐oxopentanoate.

**Table 2:** Proportion of the association of metabolites with insulin resistance and blood pressure that is mediated by their association with VF mass

Metabolite name	Trait	Metabolite *R* ^2^, no VF mass^a^	Metabolite *R* ^2^, with VF mass^b^	% Association through VF mass
**3‐methyl‐2‐oxovalerate**	HOMA‐IR	0.0126	0.0079	37.3
**4‐methyl‐2‐oxopentanoate**	HOMA‐IR	0.0031	0.0012	61.3
**Isoleucine**	HOMA‐IR	0.0162	0.0111	31.5
**Lactate**	HOMA‐IR	0.0526	0.0424	19.4
**Leucine**	HOMA‐IR	0.0073	0.0039	46.6
**Valine**	HOMA‐IR	0.0181	0.0118	34.8
**5α‐androstan‐3β, 17β‐diol disulfate‐1**	SBP	0.01	0.0076	24.0
**HWESASXX**	SBP	0.0041	0.0025	39.0
**Lactate**	SBP	0.0033	0.0022	33.3
**Palmitate**	SBP	0.0119	0.0105	11.8
**Phenylacetylglutamine**	SBP	0.0035	0.0024	31.4

a The proportion of the variance in HOMA‐IR or SBP explained by the metabolite after taking into account all covariates (age, sex, BMI, height, and metabolite batch).

b The proportion of the variance in HOMA‐IR or SBP explained by the metabolite after taking into account the same covariates as in the first footnote and adjusting also for VF mass.

HOMA‐IR, homeostasis model assessment‐estimated insulin resistance; VF, visceral fat; SBP, systolic blood pressure.

All these metabolites are indirectly related to the TCA cycle and metabolism. We assessed whether the expression of genes known to be part of the TCA cycle in subcutaneous adipose tissue correlated with VF mass. We selected human homologues for a list of TCA genes reported originally for other organisms 20, which had probes that passed quality control in our adipose tissue data. The probes used mapped to citrate synthase (*CS*); aconitase 1 (*ACO1*); isocitrate dehydrogenase 2 (*IDH2*); succinate dehydrogenase complex, subunit A (*SDHA*); succinate dehydrogenase complex, subunit B (*SDHB*), and succinate dehydrogenase complex, subunit C (*SDHC*) 20. The results (Table 3) show a strong correlation between TCA gene expression levels in fat biopsies and VF fat mass after adjusting for age and BMI. No single gene from the TCA cycle is associated with T2D, but adjusting for T2D makes the association with VF mass disappear, suggesting a confounding effect for the association between TCA cycle genes and T2D on fat mass. We also found that these genes are correlated with metabolites that associate with both VF and T2D.

**Table 3:** Tricarboxylic acid (TCA) cycle genes and VF and their association with VF and T2D metabolites

			VF		VF adj T2D,	T2D,	Metabolites
Gene	Enzyme encoded	Probe/position	*β* (SE)	*P*	*P*	*P*	(*β* [SE] with *P* < 0.05)
***CS***	Citrate synthase	ilmn_1757872 chr8:8213004:8213053	−0.0003 (0.0001)	1.32 × 10^−4^	NS	NS	Valine (−0.02 [0.01])
***ACO1***	Soluble aconitase 1	ilmn_1750800 chr9: 32440621:32440670	−0.0002 (0.0001)	1.53 × 10^−5^	NS	NS	3‐methyl‐2‐oxovalerate (−0.02 [0.01]) Isoleucine (−0.03 [0.01]) Leucine(−0.02 [0.01])
***IDH2***	Mitochondrial isocitrate dehydrogenase 2 (NADP+)	ilmn_1751753chr15:88428516:88428565	−0.0005 (0.0001)	4.04 × 10^−10^	NS	0.03	3‐methyl‐2‐oxovalerate (−0.03 [0.01]) Leucine (−0.02 [0.01]) 4‐methyl‐2‐oxopentanoate (−0.04 [0.01])
***SDHA***	Succinate dehydrogenase complex, subunit A	ilmn_1744210chr5: 289627:289676	−0.0004 (0.0001)	3.89 × 10^−5^	NS	NS	None
***SDHCB***	Succinate dehydrogenase complex, subunit C	chr1: 17221716:17221765	−0.0004 (0.0001)	1.54 × 10^−8^	NS	NS	None
***SDHC***	Succinate dehydrogenase complex, subunit C	ilmn_1667257chr1: 159601077:159601126	0.0001 (0.0001)	2.78 × 10^−2^	NS	NS	None

NS, not significant: *P* > 0.05.

VF, visceral fat; T2D, type 2 diabetes.

### Metabolites associated with T2D but not VF mass

Not all the metabolites so far reported to be associated with T2D are linked to VF mass; however, 12 metabolites including 8 lipids, 1 amino acid related to inflammation and endothelial dysfunction (dimethyl arginine), and 3 carbohydrates or metabolites of energy metabolism are associated with T2D but not with VF. Ten of these 12 are associated with a decreased risk of T2D (Figure 1). Of those associated with decreased risk of T2D and not with insulin resistance, the most biologically interesting metabolites appear to be 1,5‐anhydroglucitol, palmitoleate [previously shown be elevated in fatty liver disease and to reflect hepatic lipogenesis 21], and dimethyl arginine.

### Metabolites associated with VF mass and BP

**Figure 2:**
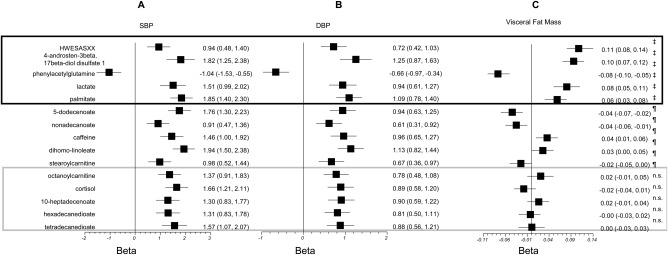
Association of selected metabolites with (**A**) systolic blood pressure (SBP), (**B**) diastolic blood pressure (DBP), and (**C**) visceral fat mass. The metabolites are those previously reported to be associated with BP with *P* < 0.0001. The linear regression coefficients β and the 95% confidence intervals of the linear regression with the three traits adjusted for covariates are shown for each metabolite. *P* values are ‡*P* < 0.0001, ¶0.0001 < *P* < 0.05, and n.s. *P* > 0.05.

In addition to the strong relationship with insulin resistance, VF mass is also a marker for higher cardiovascular risk 22. We thus carried a similar analysis comparing VF mass metabolites with those we previously found associated with SBP and DBP 9 (Figure 2). Four metabolites associated with increased BP are also associated with higher VF mass: the inflammation‐associated peptide HWESASXX, the steroid hormone androstenedione, lactate, and palmitate. One metabolite associated with lower BP, phenylacetylglutamine, was also associated with lower VF mass. As in the case of T2D, all these associations remained essentially identical when we adjusted for total fat mass. We also assessed how much of the association between these metabolites and BP was mediated by VF mass. On average, 27.9% of the variance of SBP explained by the metabolites is accounted for by the association with VF mass (Table 2), ranging from 11.8% for palmitate to 39.0% for HWESASXX.

### Metabolites associated with VF mass but not T2D

We examined significant metabolites associated with VF mass. We identified 53 metabolites significantly associated with VF after adjusting for covariates and multiple testing using Bonferroni correction. We analyzed, in a multivariate model, which of these metabolites contributed independently, and we identified 20 independent metabolites explaining 74% of the variance. As shown in Table 4, many of these metabolites are not associated with T2D nor BP. However, after adjusting for insulin resistance, only γ‐tocopherol remains significantly associated with VF.

**Table 4:** Metabolites independently associated with VF (*P* < 0.01 in backward linear regression) and their association with T2D, HOMA‐IR, and SBP

Metabolite name	Subpathway	VF, *β* (SE)	T2D, OR (SE)	HOMA‐IR, *β* (SE)	SBP, *β* (SE)
**Asparagine**	Alanine and aspartate metabolism	−0.07 (0.01)^b^	1.16 (0.15)	−0.06 (0.01)^b^	0.3 (−0.19 to 0.79)
**Creatine**	Creatine metabolism	0.08 (0.01)^c^	0.96 (0.12)	−0.03 (0.02)	−0.02 (−0.49 to 0.45)
**Glutamate**	Glutamate metabolism	0.13 (0.01)^c^	1.37 (0.19)^a^	0.14 (0.02)^c^	0.41 (−0.09 to 0.91)
**Serine**	Glycine, serine, threonine metabolism	−0.08 (0.01)^c^	0.85 (0.12)	−0.1 (0.01)^c^	0.09 (−0.4 to 0.59)
**Phenylacetylglutamine**	Phenylalanine and tyrosine metabolism	−0.08 (0.01)^c^	0.97 (0.12)	0.03 (0.01)	−1.04 (−1.53 to −0.55)^b^
**α‐Hydroxyisovalerate**	Valine, leucine, isoleucine metabolism	0.05 (0.01)^b^	1.53 (0.21)^a^	0.05 (0.02)^a^	1.31 (0.83 to 1.78)^b^
**Glycerate**	Glycolysis, gluconeogenesis, pyruvate metabolism	−0.06 (0.01)^b^	0.95 (0.09)	−0.04 (0.01)^a^	0.86 (0.35 to 1.38)^a^
**Lactate**	Glycolysis, gluconeogenesis, pyruvate metabolism	0.08 (0.01)^c^	1.84 (0.24)^b^	0.15 (0.01)^c^	1.51 (0.99 to 2.02)^b^
**γ‐Tocopherol**	Tocopherol metabolism	0.06 (0.01)^b^	0.93 (0.12)	0.03 (0.02)^a^	0.7 (0.22 to 1.17)^a^
**Hyodeoxycholate**	Bile acid metabolism	0.06 (0.01)^b^	1.47 (0.19)^a^	0.06 (0.02)^a^	
**Hexanoylcarnitine**	Carnitine metabolism	0.07 (0.01)^b^	0.6 (0.08)^a^	0.03 (0.02)^a^	1.48 (1.01 to 1.96)^c^
**1‐arachidonoylglycerophosphoinositol** a	Lysolipid	0.09 (0.01)^c^	1.06 (0.14)	0.02 (0.01)	0.61 (0.15 to 1.08)^a^
**Laurate (12:0)**	Medium‐chain fatty acid	−0.07 (0.01)^b^	0.69 (0.08)^a^	−0.03 (0.01)^a^	1.52 (1.04 to 2)^c^
**5α‐androstan‐3β,17β‐diol disulfate**	Sterol/steroid	0.09 (0.01)^c^	1.28 (0.2)	0.04 (0.02)^a^	
**Urate**	Purine and urate metabolism	0.11 (0.01)^c^	0.92 (0.13)	0.1 (0.02)^c^	1.09 (0.57 to 1.6)^b^
**Cyclo(leu‐pro)**	Dipeptide	0.06 (0.02)^b^	1.16 (0.14)	0.08 (0.02)^b^	
**γ‐glutamylvaline**	γ‐glutamyl	0.11 (0.01)^c^	1.41 (0.17)^a^	0.15 (0.02)^c^	0.45 (−0.04 to 0.94)^a^
**HWESASXX** a	Polypeptide	0.11 (0.01)^c^	1.28 (0.15)^a^	0.09 (0.02)^b^	0.94 (0.48 to 1.4)^b^
**Catechol sulfate**	Benzoate metabolism	−0.07 (0.01)^b^	1.2 (0.15)	0 (0.01)	−0.61 (−1.06 to −0.16)^a^
**Stachydrine**	Food component/plant	−0.06 (0.01)^b^	1.01 (0.12)	−0.03 (0.02)	−0.09 (−0.56 to 0.37)

Data are presented as coefficient from linear regression (*β*) and standard error (SE) or as odds ratio (OR) and standard error.

a *P* < 0.05.

b *P* < 0.0001.

c *P* < 1 × 10^−8^.

HOMA‐IR, homeostasis model assessment‐estimated insulin resistance; VF, visceral fat; T2D, type 2 diabetes; SBP, systolic blood pressure.

**Figure 3:**
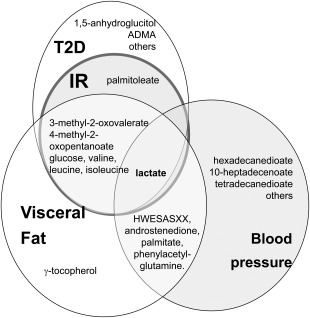
Venn diagram summarizing which metabolites are associated with visceral fat (VF), insulin resistance (IR), blood pressure (BP), and type 2 diabetes (T2D) in this study.

A summary of the results is presented in a Venn diagram, showing the key metabolites associated with insulin resistance, VF, BP, and T2D (Figure 3).

## Discussion

In the present metabolomic study, we investigated which metabolites associated with VF mass relate also to insulin resistance/T2D and BP. We identified seven metabolites that strongly associated with both T2D and VF mass and whose association with insulin resistance appears to be in part mediated by VF mass. These include glucose and three BCAAs. We also identified 12 metabolites (8 lipids, one amino acid, 2 carbohydrates, and 1 intermediate of energy metabolism) that show no correlation with VF mass despite their strong association with T2D. Finally we identified five metabolites in common between BP and VF mass.

### Metabolites associated with both T2D and VF mass

All the metabolites associated with both VF and T2D appear to be related to central energy metabolism, which also regulates the intracellular ratio of nicotinamide adenine dinucleotide hydrogenase/nicotinamide adenine dinucleotide levels. Lactate is produced from pyruvate under anaerobic conditions with the concomitant oxidation of NADH to NAD. The BCAAs valine, leucine, isoleucine are essential amino acids (not synthesized by humans), hence their concentration in blood is caused by a lack of catabolism. BCAAs are converted by transamination into BCKAs 23, two of which (3‐methyl‐2‐oxovalerate and 4‐methyl‐2‐oxopentanoate) were also increased in individuals with high VF mass. When energy metabolism is properly functioning, these metabolites should be oxidised via the mitochondrial branched‐chain keto acid dehydrogenase complex to CO_2_, NADH, and ultimately produce acetyl‐CoA and succinyl‐CoA that enter the TCA cycle producing large mole to mole equivalents of ATP (e.g. 39 moles ATP/mole leucine). However, the branched‐chain keto acid dehydrogenase complex is subject to feedback regulation 24, which explains the accumulation of BCAAs and their metabolites. Other studies have previously shown that BCAAs are significantly correlated with a metabolically unhealthy phenotype, and gene expression of BCAA catabolism and TCA cycle‐related genes are underexpressed in metabolically unhealthy individuals compared to healthy subjects 25.

A reduced TCA cycle has been invoked as a maker of the diabetic phenotype 26, and our data suggest that some of these disturbances of the TCA cycle are mediated by VF mass. The gene expression results presented here show a clear negative relationship between genes encoding TCA cycle enzymes in adipose tissue and VF mass, and this association goes away once we adjust for T2D. Since abdominal obesity is modifiable, based on the current data we hypothesize that reductions in VF mass should result in improved TCA cycle efficiency in patients with T2D.

### Metabolites associated with VF mass and BP

We identified four metabolites associated with increased BP and also with higher VF mass: the inflammation‐associated peptide HWESASXX, the steroid hormone androstenedione, lactate, and palmitate. On average 28.9% of their association with SBP appears to be mediated by VF mass. (Table 2). We also identified one metabolite, phenylacetylglutamine, associated with lower BP and lower VF mass.

Only lactate is associated with VF mass, insulin resistance, and BP. Lactate's metabolism is not only altered in obesity 27, but metabolic shift to aerobic glycolysis, both in pulmonary vasculature and the right ventricle is seen in pulmonary HTN similar to the Warburg effect seen in cancer cells 28. It is possible that a similar mechanism combined with obesity may be at work in arterial HTN, and a considerable proportion of this effect is mediated by VF. Whereas less than 20% of the effect of lactate on insulin resistance can be explained by its effect on VF, 33% of its effect on SBP can be explained by its effect on VF (Table 2). The pivotal role of lactate highlights the importance of energy metabolism as the key link between increased VF and higher risk of metabolic syndrome.

The strongest contribution via VF on SBP was seen, however, with HWESASXX (39%). This peptide is linked to inflammation 29 and is known to increase some of the biological actions of insulin‐like growth factors 30. This peptide has also been linked to intake of antihypertensive drugs 31, hence the association with SBP could have resulted from use of antihypertensive medication in those with higher BP. However, the association between HWESASXX and SBP remains significant after excluding individuals with a diagnosis of HTN (*β*(SE) = 0.99 (0.23), *P* = 1.3 × 10^−5^), so it appears to be independent of medication use. The second metabolite identified, androstenedione (4‐androstenedione, 17‐ketotestosterone) is a steroid hormone 30. Increased levels of this compound have been reported in childhood obesity, with adiposity and worse cardiometabolic profile 32. Palmitic acid is the first fatty acid produced during lipogenesis. In humans, increases in dietary palmitate result in a decrease in fat oxidation and daily energy expenditure, indicating that it increases the risk of obesity and insulin resistance 33. This is further supported by our own gene expression data which shows that palmitate circulating levels are negatively correlated with peroxisome proliferator‐activated receptor‐γ gene expression levels in adipose tissue (*β* = 0.08 (0.01) *P =* 2.90 × 10^−9^). A diet rich in palmitic acid has been demonstrated to result in a BP‐dependent increase in arterial stiffness 34. However, less than 12% of the effect of palmitic acid on SBP is due to its effect on VF mass.

Finally, the only compound associated significantly with both lower BP and lower VF mass is phenylacetylglutamine. We have previously reported the protective role of phenylacetylglutamine on arterial stiffness and Framingham cardiovascular risk in this same cohort 35 and the negative correlation with VF indicates that this molecule is a marker or is involved in lower risk of metabolic syndrome.

### Metabolites associated with T2D but not VF mass

We identified 12 metabolites associated with T2D but not with VF, indicating that although VF mass has an important contribution to the molecular pathogenesis of T2D, there are a number of molecular markers strongly linked to T2D that are independent of VF mass. The most significant metabolite associated with T2D but not associated with VF is 1,5‐anhydroglucitol, a well‐known marker of short‐term glycemic control inversely proportional to glucose and is currently proposed as a nontraditional marker in diabetes diagnosis, prognosis, and management [see Parrinello and Selvin for review 36].

We note some study limitations. First, the study sample consists of women only, and some metabolites might be influenced by sex‐specific hormones. However, many of the metabolites identified in this female‐only sample with regards to T2D, specifically the BCAAs, have been also identified in other studies where both men and women were included 8, 37 as associated with insulin resistance and obesity, supporting the generalization of our results. Second, because DXA measurements are not available in other Metabolon metabolomics cohorts, we could not validate our VF results in an independent sample. Furthermore, the cross‐sectional nature of our data does not allow us to draw conclusions as to whether the metabolites identified are causative of VF/T2D or merely correlated with it. However, our results shed light on the TCA cycle as a key pathway linking VF mass to T2D risk, which deserves further investigation. Finally, the observations derived from the mediation approach shown here (proportion of association of metabolites due to VF) are of purely statistical nature and do not necessarily imply a functional connection.

## Conclusion

The current study shows that of the metabolites associated with T2D, those related to VF are all linked to energy metabolism, in particular to glycolysis and the TCA cycle, and that on average 39% of the association of these metabolites with insulin resistance is mediated by their association with VF mass. We hypothesize that reductions in VF mass may potentially result in improving the mitochondrial TCA cycle efficiency seen in patients with diabetes. This is consistent with a recent therapeutic trial reporting that a very‐low‐calorie diet can achieve nondiabetic fasting glucose levels in 50% of patients while remaining off all other antidiabetic therapies 38. The link between VF mass and BP appears to be related to energy metabolism as well, via lactate and FFAs, but we find also a link with steroids and inflammation.

## Supporting information

Supporting Information

## References

[1] Finelli C , Sommella L , Gioia S , La Sala N , Tarantino G. Should visceral fat be reduced to increase longevity? Ageing Res Rev 2013;12:996‐1004. 23764746 10.1016/j.arr.2013.05.007

[2] Kershaw EE , Flier JS. Adipose tissue as an endocrine organ. J Clin Endocrinol Metab 2004;89:2548‐2556. 15181022 10.1210/jc.2004-0395

[3] Floegel A , Stefan N , Yu Z , et al. Identification of serum metabolites associated with risk of type 2 diabetes using a targeted metabolomic approach. Diabetes 2013;62:639‐648. 23043162 10.2337/db12-0495 PMC3554384

[4] Gall WE , Beebe K , Lawton KA , et al. alpha‐hydroxybutyrate is an early biomarker of insulin resistance and glucose intolerance in a nondiabetic population. PLoS One 2010;5:e10883. 20526369 10.1371/journal.pone.0010883 PMC2878333

[5] Menni C , Fauman E , Erte I , et al. Biomarkers for type 2 diabetes and impaired fasting glucose using a nontargeted metabolomics approach. Diabetes 2013;62:4270‐4276. 23884885 10.2337/db13-0570 PMC3837024

[6] Suhre K , Meisinger C , Doring A , et al. Metabolic footprint of diabetes: a multiplatform metabolomics study in an epidemiological setting. PLoS One 2010;5:e13953. 21085649 10.1371/journal.pone.0013953 PMC2978704

[7] Wang TJ , Larson MG , Vasan RS , et al. Metabolite profiles and the risk of developing diabetes. Nat Med 2011;17:448‐453. 21423183 10.1038/nm.2307 PMC3126616

[8] Wurtz P , Tiainen M , Makinen VP , et al. Circulating metabolite predictors of glycemia in middle‐aged men and women. Diabetes Care 2012;35:1749‐1756. 22563043 10.2337/dc11-1838 PMC3402262

[9] Menni C , Graham D , Kastenmuller G , et al. Metabolomic identification of a novel pathway of blood pressure regulation involving hexadecanedioate. Hypertension 2015;66:422–429. 26034203 10.1161/HYPERTENSIONAHA.115.05544 PMC4490909

[10] Ebbert JO , Jensen MD. Fat depots, free fatty acids, and dyslipidemia. Nutrients 2013;5:498‐508. 23434905 10.3390/nu5020498 PMC3635208

[11] Klein S. The case of visceral fat: argument for the defense. J Clin Invest 2004;113:1530‐1532. 15173878 10.1172/JCI22028 PMC419497

[12] Su X , Abumrad NA. Cellular fatty acid uptake: a pathway under construction. Trends Endocrinol Metabol 2009;20:72‐77. 10.1016/j.tem.2008.11.001 PMC2845711 19185504

[13] Fiehn O , Garvey WT , Newman JW , Lok KH , Hoppel CL , Adams SH. Plasma metabolomic profiles reflective of glucose homeostasis in non‐diabetic and type 2 diabetic obese African‐American women. PLoS One 2010;5:e15234. 21170321 10.1371/journal.pone.0015234 PMC3000813

[14] Moayyeri A , Hammond CJ , Valdes AM , Spector TD. Cohort profile: TwinsUK and healthy ageing twin study. Int J Epidemiol 2013;42:76‐85. 22253318 10.1093/ije/dyr207 PMC3600616

[15] Kaul S , Rothney MP , Peters DM , et al. Dual‐energy X‐ray absorptiometry for quantification of visceral fat. Obesity (Silver Spring) 2012;20:1313‐1318. 22282048 10.1038/oby.2011.393 PMC3361068

[16] Falchi M , Wilson SG , Paximadas D , Swaminathan R , Spector TD. Quantitative linkage analysis for pancreatic B‐cell function and insulin resistance in a large twin cohort. Diabetes 2008;57:1120‐1124. 18174525 10.2337/db07-0708

[17] Matthews DR , Hosker JP , Rudenski AS , Naylor BA , Treacher DF , Turner RC. Homeostasis model assessment: insulin resistance and beta‐cell function from fasting plasma glucose and insulin concentrations in man. Diabetologia 1985;28:412‐419. 3899825 10.1007/BF00280883

[18] Menni C , Kastenmuller G , Petersen AK , et al. Metabolomic markers reveal novel pathways of ageing and early development in human populations. Int J Epidemiol 2013;42:1111‐1119. 23838602 10.1093/ije/dyt094 PMC3781000

[19] Grundberg E , Small KS , Hedman AK , et al. Mapping cis‐ and trans‐regulatory effects across multiple tissues in twins. Nat Genet 2012;44:1084‐1089. 22941192 10.1038/ng.2394 PMC3784328

[20] Przybyla‐Zawislak B , Gadde DM , Ducharme K , McCammon MT. Genetic and biochemical interactions involving tricarboxylic acid cycle (TCA) function using a collection of mutants defective in all TCA cycle genes. Genetics 1999;152:153‐166. 10224250 10.1093/genetics/152.1.153 PMC1460613

[21] Lee JJ , Lambert JE , Hovhannisyan Y , et al. Palmitoleic acid is elevated in fatty liver disease and reflects hepatic lipogenesis. Am J Clin Nutr 2015;101:34‐43. 25527748 10.3945/ajcn.114.092262 PMC4266891

[22] Vaneckova I , Maletinska L , Behuliak M , Nagelova V , Zicha J , Kunes J. Obesity‐related hypertension: possible pathophysiological mechanisms. J Endocrinol 2014;223:R63‐R78. 25385879 10.1530/JOE-14-0368

[23] Brosnan JT , Brosnan ME. Branched‐chain amino acids: enzyme and substrate regulation. J Nutr 2006;136:207S‐211S. 16365084 10.1093/jn/136.1.207S

[24] Sears DD , Hsiao G , Hsiao A , et al. Mechanisms of human insulin resistance and thiazolidinedione‐mediated insulin sensitization. Proc Natl Acad Sci USA 2009;106:18745‐18750. 19841271 10.1073/pnas.0903032106 PMC2763882

[25] Badoud F , Lam KP , DiBattista A , et al. Serum and adipose tissue amino acid homeostasis in the metabolically healthy obese. J Proteome Res 2014;13:3455‐3466. 24933025 10.1021/pr500416v

[26] Gaster M , Nehlin JO , Minet AD. Impaired TCA cycle flux in mitochondria in skeletal muscle from type 2 diabetic subjects: marker or maker of the diabetic phenotype? Arch Physiol Biochem 2012;118:156‐189. 22385297 10.3109/13813455.2012.656653

[27] Lovejoy J , Newby FD , Gebhart SS , DiGirolamo M. Insulin resistance in obesity is associated with elevated basal lactate levels and diminished lactate appearance following intravenous glucose and insulin. Metabolism 1992;41:22‐27. 1538640 10.1016/0026-0495(92)90185-d

[28] Tuder RM , Davis LA , Graham BB. Targeting energetic metabolism: a new frontier in the pathogenesis and treatment of pulmonary hypertension. Am J Respir Crit Care Med 2012;185:260‐266. 22077069 10.1164/rccm.201108-1536PP PMC3297113

[29] Finkelstein JL , Pressman EK , Cooper EM , Kent TR , Bar HY , O'Brien KO , Vitamin D. Status affects serum metabolomic profiles in pregnant adolescents. Reprod Sci 2014;22(6):422–429. 10.1177/1933719114556477 PMC4502797 25367051

[30] Dousset B , Straczek J , Maachi F , et al. Purification from human plasma of a hexapeptide that potentiates the sulfation and mitogenic activities of insulin‐like growth factors. Biochem Biophys Res Commun 1998;247:587‐591. 9647737 10.1006/bbrc.1998.8834

[31] Altmaier E , Fobo G , Heier M , et al. Metabolomics approach reveals effects of antihypertensives and lipid‐lowering drugs on the human metabolism. Eur J Epidemiol 2014;29:325‐336. 24816436 10.1007/s10654-014-9910-7 PMC4050296

[32] Perng W , Gillman MW , Fleisch AF , et al. Metabolomic profiles and childhood obesity. Obesity (Silver Spring) 2014;22:2570‐2578. 25251340 10.1002/oby.20901 PMC4236243

[33] Kien CL , Bunn JY , Ugrasbul F. Increasing dietary palmitic acid decreases fat oxidation and daily energy expenditure. Am J Clin Nutr 2005;82:320‐326. 16087974 10.1093/ajcn.82.2.320 PMC1314972

[34] Lithander FE , Herlihy LK , Walsh DM , Burke E , Crowley V , Mahmud A. Postprandial effect of dietary fat quantity and quality on arterial stiffness and wave reflection: a randomised controlled trial. Nutr J 2013;12:93 23841960 10.1186/1475-2891-12-93 PMC3717051

[35] Menni C , Mangino M , Cecelja M , et al. Metabolomic study of carotid‐femoral pulse‐wave velocity in women. J Hypertens 2015;33:791‐796. 25490711 10.1097/HJH.0000000000000467 PMC4354457

[36] Parrinello CM , Selvin E. Beyond HbA1c and glucose: the role of nontraditional glycemic markers in diabetes diagnosis, prognosis, and management. Curr Diabetes Rep 2014;14:548 10.1007/s11892-014-0548-3 PMC4214073 25249070

[37] Newgard CB , An J , Bain JR , et al. A branched‐chain amino acid‐related metabolic signature that differentiates obese and lean humans and contributes to insulin resistance. Cell Metab 2009;9:311‐326. 19356713 10.1016/j.cmet.2009.02.002 PMC3640280

[38] Steven S , Taylor R. Restoring normoglycaemia by use of a very low calorie diet in long‐ and short‐duration Type 2 diabetes. Diabet Med 2015;32(9):1149–1155. 25683066 10.1111/dme.12722

