# Supplementary material for: Metabolomic profiling to dissect the role of visceral fat in cardiometabolic health
Source: Obesity (Silver Spring). 2016 Apr 30;24(6):1380–8. doi: 10.1002/oby.21488 (PMC4914926; doi:10.1002/oby.21488)
Supplement: Supplementary file 1 — Supporting Information [file OBY-24-1380-s001.docx]

**Table S1. List of significant metabolites with T2D and VF in one or more comparison and their association with log HOMA-IR and lean mass**

|  | **Super-p** | **Sub-p** | **T2D** |  | **VF** | | **HOMA-IR** | | **Lean Mass** | |
| --- | --- | --- | --- | --- | --- | --- | --- | --- | --- | --- |
| **Metabolite name** |  |  | **OR(SE)** | **p** | **Beta(SE)** | **P** | **Beta(SE)** | **P** | **Beta(SE)** | **P** |
| asparagine | a-a | Alanine and aspartate metabolism | 1.16(0.15) | 2.40E-01 | -0.07(0.01) | 1.77E-08 | -0.06(0.01) | 3.25E-06 | 0.05(0.01) | 1.16E-04 |
| aspartate | a-a | Alanine and aspartate metabolism | 1.38(0.17) | 8.96E-03 | 0.11(0.01) | 1.23E-15 | 0.11(0.02) | 2.58E-13 | -0.02(0.01) | 2.68E-01 |
| creatine | a-a | Creatine metabolism | 0.96(0.12) | 7.35E-01 | 0.08(0.01) | 2.12E-09 | -0.03(0.02) | 1.35E-01 | -0.11(0.01) | 5.32E-14 |
| 2-hydroxybutyrate (AHB) | a-a | Cysteine, methionine, SAM, taurine metabolism | 1.62(0.21) | 1.63E-04 | 0.1(0.01) | 6.89E-12 | 0.02(0.02) | 1.21E-01 | -0.06(0.01) | 9.62E-05 |
| glutamate | a-a | Glutamate metabolism | 1.37(0.19) | 2.19E-02 | 0.13(0.01) | 5.53E-23 | 0.14(0.02) | 1.23E-16 | -0.03(0.02) | 6.77E-02 |
| betaine | a-a | Glycine, serine and threonine metabolism | 0.83(0.09) | 9.88E-02 | -0.05(0.01) | 8.88E-05 | -0.08(0.02) | 2.29E-07 | 0(0.01) | 7.51E-01 |
| glycine | a-a | Glycine, serine and threonine metabolism | 0.7(0.12) | 4.22E-02 | -0.08(0.01) | 1.72E-09 | -0.08(0.02) | 9.16E-08 | 0.05(0.01) | 1.40E-04 |
| N-acetylglycine | a-a | Glycine, serine and threonine metabolism | 0.59(0.08) | 6.26E-05 | -0.05(0.01) | 1.18E-04 | -0.15(0.01) | 3.77E-28 | 0.03(0.01) | 3.83E-02 |
| serine | a-a | Glycine, serine and threonine metabolism | 0.85(0.12) | 2.41E-01 | -0.08(0.01) | 1.03E-10 | -0.1(0.01) | 5.91E-12 | 0.03(0.01) | 6.17E-02 |
| 3-phenylpropionate (hydrocinnamate) | a-a | Phenylalanine & tyrosine metabolism | 1.07(0.13) | 5.46E-01 | -0.08(0.01) | 2.72E-09 | -0.02(0.02) | 1.23E-01 | 0.09(0.01) | 1.11E-09 |
| p-cresol sulfate | a-a | Phenylalanine & tyrosine metabolism | 0.94(0.11) | 6.02E-01 | -0.07(0.01) | 4.83E-08 | 0.02(0.02) | 1.98E-01 | 0.01(0.01) | 3.98E-01 |
| phenylacetate | a-a | Phenylalanine & tyrosine metabolism | 1.13(0.16) | 3.84E-01 | -0.06(0.01) | 3.79E-05 | 0(0.02) | 8.81E-01 | 0.01(0.02) | 6.34E-01 |
| phenylacetylglutamine | a-a | Phenylalanine & tyrosine metabolism | 0.97(0.12) | 7.87E-01 | -0.08(0.01) | 2.29E-09 | 0.03(0.01) | 6.07E-02 | 0.01(0.01) | 3.09E-01 |
| tyrosine | a-a | Phenylalanine & tyrosine metabolism | 1.19(0.15) | 1.73E-01 | 0.06(0.01) | 6.57E-05 | 0.09(0.02) | 4.25E-08 | 0.03(0.01) | 5.84E-02 |
| indolepropionate | a-a | Tryptophan metabolism | 1.01(0.11) | 9.24E-01 | -0.06(0.01) | 1.88E-05 | -0.05(0.02) | 3.15E-03 | 0.06(0.01) | 3.37E-05 |
| citrulline | a-a | Urea cycle; arginine-, proline-, metabolism | 0.54(0.07) | 6.14E-06 | -0.04(0.01) | 1.94E-03 | -0.04(0.02) | 1.14E-02 | 0.02(0.01) | 1.02E-01 |
| dimethylarginine (SDMA + ADMA) | a-a | Urea cycle; arginine-, proline-, metabolism | 0.55(0.08) | 5.70E-05 | -0.01(0.01) | 3.97E-01 | -0.02(0.01) | 1.18E-01 | -0.03(0.01) | 6.26E-02 |
| proline | a-a | Urea cycle; arginine-, proline-, metabolism | 1.86(0.26) | 5.85E-06 | 0.05(0.01) | 2.09E-04 | 0.09(0.02) | 2.02E-08 | -0.01(0.01) | 5.47E-01 |
| 3-methyl-2-oxobutyrate | a-a | Valine, leucine and isoleucine metabolism | 1.45(0.17) | 1.96E-03 | 0.07(0.01) | 7.89E-07 | 0.07(0.02) | 4.17E-04 | 0.02(0.01) | 1.37E-01 |
| 3-methyl-2-oxovalerate | a-a | Valine, leucine and isoleucine metabolism | 2.18(0.29) | 7.17E-09 | 0.07(0.01) | 3.30E-07 | 0.06(0.02) | 1.98E-04 | 0.04(0.01) | 3.47E-03 |
| 4-methyl-2-oxopentanoate | a-a | Valine, leucine and isoleucine metabolism | 2(0.27) | 2.04E-07 | 0.06(0.01) | 2.28E-05 | 0.02(0.02) | 3.73E-01 | 0.06(0.01) | 2.02E-05 |
| alpha-hydroxyisovalerate | a-a | Valine, leucine and isoleucine metabolism | 1.53(0.21) | 1.86E-03 | 0.05(0.01) | 3.28E-05 | 0.05(0.02) | 4.21E-04 | 0.01(0.01) | 3.45E-01 |
| isoleucine | a-a | Valine, leucine and isoleucine metabolism | 2.35(0.32) | 6.31E-10 | 0.08(0.01) | 1.53E-08 | 0.07(0.02) | 3.35E-06 | 0.03(0.01) | 6.62E-02 |
| isovalerylcarnitine | a-a | Valine, leucine and isoleucine metabolism | 1.49(0.2) | 3.01E-03 | 0.06(0.01) | 1.71E-06 | 0.08(0.01) | 1.86E-07 | 0.02(0.01) | 1.57E-01 |
| leucine | a-a | Valine, leucine and isoleucine metabolism | 2.2(0.29) | 1.99E-09 | 0.08(0.01) | 1.33E-09 | 0.04(0.02) | 3.25E-02 | 0.04(0.01) | 8.41E-03 |
| valine | a-a | Valine, leucine and isoleucine metabolism | 2.19(0.31) | 3.42E-08 | 0.08(0.01) | 5.28E-10 | 0.09(0.02) | 3.34E-08 | 0.02(0.01) | 2.94E-01 |
| fructose | ch | Fructose, mannose, galactose, starch, and sucrose metabolism | 2.21(0.31) | 2.14E-08 | -0.03(0.01) | 9.21E-03 | 0(0.01) | 9.24E-01 | 0.02(0.01) | 1.57E-01 |
| mannose | ch | Fructose, mannose, galactose, starch, and sucrose metabolism | 3.36(0.55) | 9.83E-14 | 0.03(0.01) | 2.59E-02 | 0.09(0.01) | 3.52E-10 | 0.03(0.01) | 2.04E-02 |
| 1,5-anhydroglucitol (1,5-AG) | ch | Glycolysis, gluconeogenesis, pyruvate metabolism | 0.38(0.06) | 2.42E-11 | 0.01(0.01) | 3.35E-01 | 0(0.01) | 7.68E-01 | -0.06(0.01) | 6.53E-05 |
| glucose | ch | Glycolysis, gluconeogenesis, pyruvate metabolism | 5.5(1.22) | 1.12E-14 | 0.05(0.01) | 7.19E-05 | 0.11(0.01) | 1.47E-14 | 0.04(0.01) | 6.96E-03 |
| glycerate | ch | Glycolysis, gluconeogenesis, pyruvate metabolism | 0.95(0.09) | 5.74E-01 | -0.06(0.01) | 9.37E-06 | -0.04(0.01) | 7.04E-03 | 0.05(0.01) | 7.61E-04 |
| lactate | ch | Glycolysis, gluconeogenesis, pyruvate metabolism | 1.84(0.24) | 2.11E-06 | 0.08(0.01) | 5.03E-09 | 0.15(0.01) | 2.55E-22 | -0.01(0.02) | 3.21E-01 |
| arabinose | ch | Nucleotide sugars, pentose metabolism | 2.07(0.34) | 8.31E-06 | -0.01(0.01) | 6.82E-01 | 0.04(0.02) | 2.17E-02 | -0.02(0.01) | 2.62E-01 |
| gamma-tocopherol | c&v | Tocopherol metabolism | 0.93(0.12) | 6.02E-01 | 0.06(0.01) | 1.26E-05 | 0.03(0.02) | 4.51E-02 | 0(0.01) | 8.14E-01 |
| citrate | e | Krebs cycle | 0.96(0.11) | 7.02E-01 | -0.06(0.01) | 4.88E-06 | -0.04(0.01) | 5.07E-03 | 0.02(0.02) | 2.38E-01 |
| malate | e | Krebs cycle | 1.63(0.2) | 9.12E-05 | 0.01(0.01) | 4.72E-01 | 0.04(0.02) | 3.40E-03 | 0.03(0.01) | 3.90E-02 |
| hyodeoxycholate | l | Bile acid metabolism | 1.47(0.19) | 3.20E-03 | 0.06(0.01) | 1.67E-05 | 0.06(0.02) | 3.22E-04 | -0.04(0.02) | 1.47E-02 |
| carnitine | l | Carnitine metabolism | 1.04(0.14) | 7.75E-01 | 0.09(0.01) | 5.00E-11 | 0.04(0.02) | 4.78E-03 | -0.01(0.01) | 3.31E-01 |
| hexanoylcarnitine | l | Carnitine metabolism | 0.6(0.08) | 1.27E-04 | 0.07(0.01) | 1.07E-06 | 0.03(0.02) | 4.85E-02 | -0.08(0.02) | 4.41E-07 |
| octanoylcarnitine | l | Carnitine metabolism | 0.62(0.08) | 9.88E-05 | 0.02(0.01) | 1.21E-01 | 0.02(0.02) | 1.27E-01 | -0.05(0.02) | 1.57E-03 |
| dihomo-linolenate (20:3n3 or n6) | l | Essential fatty acid | 0.83(0.1) | 1.15E-01 | 0.05(0.01) | 9.65E-05 | 0.01(0.01) | 4.33E-01 | -0.06(0.01) | 3.28E-05 |
| butyrylcarnitine | l | Fatty acid metabolism (also BCAA metabolism) | 0.92(0.12) | 5.40E-01 | 0.08(0.01) | 2.47E-09 | 0.09(0.02) | 1.33E-07 | -0.07(0.02) | 1.19E-06 |
| propionylcarnitine | l | Fatty acid metabolism (also BCAA metabolism) | 1.39(0.17) | 5.52E-03 | 0.06(0.01) | 8.29E-06 | 0.09(0.02) | 4.13E-09 | -0.01(0.02) | 5.05E-01 |
| 15-methylpalmitate (isobar with 2-methylpalmitate) | l | Fatty acid, branched | 0.61(0.07) | 4.77E-05 | -0.01(0.01) | 6.25E-01 | -0.02(0.01) | 2.37E-01 | -0.02(0.01) | 2.18E-01 |
| 10-heptadecenoate (17:1n7) | l | Long chain fatty acid | 0.56(0.07) | 2.31E-06 | 0.02(0.01) | 2.44E-01 | -0.04(0.02) | 2.97E-02 | -0.07(0.02) | 4.40E-06 |
| adrenate (22:4n6) | l | Long chain fatty acid | 0.95(0.13) | 7.16E-01 | 0.06(0.01) | 4.45E-07 | 0.03(0.01) | 5.94E-02 | -0.06(0.01) | 4.06E-06 |
| arachidonate (20:4n6) | l | Long chain fatty acid | 0.91(0.11) | 4.56E-01 | 0.07(0.01) | 2.03E-07 | 0.01(0.01) | 4.61E-01 | -0.06(0.01) | 5.77E-05 |
| myristate (14:0) | l | Long chain fatty acid | 0.64(0.07) | 6.30E-05 | -0.01(0.01) | 2.88E-01 | -0.02(0.02) | 3.07E-01 | -0.02(0.01) | 1.33E-01 |
| myristoleate (14:1n5) | l | Long chain fatty acid | 0.44(0.06) | 1.22E-10 | -0.04(0.01) | 9.56E-03 | -0.06(0.02) | 9.15E-05 | -0.06(0.02) | 1.90E-05 |
| palmitate (16:0) | l | Long chain fatty acid | 0.8(0.09) | 5.65E-02 | 0.06(0.01) | 2.03E-05 | 0.01(0.02) | 5.91E-01 | -0.05(0.01) | 2.83E-04 |
| palmitoleate (16:1n7) | l | Long chain fatty acid | 0.5(0.06) | 5.10E-08 | 0.01(0.01) | 5.02E-01 | -0.06(0.02) | 9.19E-05 | -0.08(0.01) | 9.35E-08 |
| pentadecanoate (15:0) | l | Long chain fatty acid | 0.65(0.07) | 7.02E-05 | -0.02(0.01) | 2.06E-01 | -0.01(0.01) | 3.33E-01 | -0.01(0.01) | 6.06E-01 |
| 1-arachidonoylglycerophosphoinositol* | l | Lysolipid | 1.06(0.14) | 6.70E-01 | 0.09(0.01) | 3.41E-13 | 0.02(0.01) | 9.19E-02 | -0.07(0.01) | 1.51E-07 |
| 1-eicosatrienoylglycerophosphocholine* | l | Lysolipid | 0.83(0.11) | 1.55E-01 | 0.05(0.01) | 2.32E-05 | -0.03(0.01) | 5.98E-02 | -0.02(0.01) | 8.11E-02 |
| 5-dodecenoate (12:1n7) | l | Medium chain fatty acid | 0.43(0.06) | 1.02E-10 | -0.04(0.01) | 8.37E-04 | -0.06(0.02) | 6.87E-05 | -0.06(0.01) | 3.63E-05 |
| heptanoate (7:0) | l | Medium chain fatty acid | 0.53(0.07) | 2.14E-06 | -0.02(0.01) | 1.59E-01 | -0.08(0.02) | 2.32E-06 | -0.03(0.01) | 3.56E-02 |
| laurate (12:0) | l | Medium chain fatty acid | 0.69(0.08) | 9.82E-04 | -0.07(0.01) | 6.69E-07 | -0.03(0.01) | 1.82E-02 | -0.02(0.01) | 7.91E-02 |
| pelargonate (9:0) | l | Medium chain fatty acid | 0.57(0.08) | 5.54E-05 | -0.01(0.01) | 5.10E-01 | -0.08(0.02) | 2.01E-06 | -0.02(0.01) | 2.04E-01 |
| palmitoyl sphingomyelin | l | Sphingolipid | 0.44(0.06) | 5.30E-10 | -0.05(0.01) | 1.38E-04 | -0.11(0.02) | 1.89E-12 | -0.02(0.01) | 2.12E-01 |
| 4-androsten-3beta,17beta-diol disulfate 1* | l | Sterol/Steroid | 1.03(0.15) | 8.58E-01 | 0.1(0.01) | 1.90E-11 | 0.03(0.02) | 3.05E-02 | 0.03(0.01) | 4.65E-02 |
| 5alpha-androstan-3beta,17beta-diol disulfate | l | Sterol/Steroid | 1.28(0.2) | 1.14E-01 | 0.09(0.01) | 2.72E-11 | 0.04(0.02) | 2.39E-02 | 0(0.01) | 7.39E-01 |
| cholesterol | l | Sterol/Steroid | 0.52(0.07) | 1.25E-06 | 0.03(0.01) | 4.67E-02 | -0.05(0.02) | 2.31E-03 | -0.02(0.01) | 1.88E-01 |
| lathosterol | l | Sterol/Steroid | 0.72(0.09) | 1.15E-02 | 0.06(0.01) | 1.48E-05 | 0.06(0.02) | 3.88E-04 | 0(0.02) | 7.59E-01 |
| urate | n | Purine metabolism, urate metabolism | 0.92(0.13) | 5.79E-01 | 0.11(0.01) | 5.27E-16 | 0.1(0.02) | 6.40E-11 | -0.03(0.02) | 2.56E-02 |
| cyclo(leu-pro) | p | Dipeptide | 1.16(0.14) | 2.21E-01 | 0.06(0.02) | 8.09E-05 | 0.08(0.02) | 1.93E-05 | 0.01(0.02) | 6.53E-01 |
| gamma-glutamylisoleucine* | p | gamma-glutamyl | 1.44(0.18) | 3.06E-03 | 0.09(0.02) | 5.47E-08 | 0.12(0.02) | 9.36E-07 | -0.01(0.02) | 4.08E-01 |
| gamma-glutamylleucine | p | gamma-glutamyl | 1.55(0.19) | 2.26E-04 | 0.09(0.01) | 2.63E-10 | 0.1(0.02) | 3.57E-08 | 0.01(0.02) | 6.47E-01 |
| gamma-glutamylphenylalanine | p | gamma-glutamyl | 1.08(0.14) | 5.59E-01 | 0.06(0.01) | 3.01E-05 | 0.08(0.02) | 5.80E-07 | -0.01(0.01) | 6.76E-01 |
| gamma-glutamyltyrosine | p | gamma-glutamyl | 0.94(0.12) | 6.17E-01 | 0.07(0.01) | 8.72E-06 | 0.1(0.02) | 2.75E-08 | -0.01(0.02) | 5.72E-01 |
| gamma-glutamylvaline | p | gamma-glutamyl | 1.41(0.17) | 5.04E-03 | 0.11(0.01) | 3.32E-13 | 0.15(0.02) | 6.56E-15 | -0.02(0.02) | 3.05E-01 |
| HWESASXX* | p | Polypeptide | 1.28(0.15) | 3.79E-02 | 0.11(0.01) | 4.07E-13 | 0.09(0.02) | 5.63E-07 | -0.04(0.01) | 4.27E-03 |
| catechol sulfate | x | Benzoate metabolism | 1.2(0.15) | 1.47E-01 | -0.07(0.01) | 4.02E-08 | 0(0.01) | 9.40E-01 | 0.05(0.01) | 8.83E-05 |
| hippurate | x | Benzoate metabolism | 1.03(0.12) | 8.28E-01 | -0.09(0.01) | 2.56E-12 | -0.02(0.01) | 3.03E-01 | 0.07(0.01) | 5.26E-07 |
| stachydrine | x | Food component/Plant | 1.01(0.12) | 9.60E-01 | -0.06(0.01) | 1.13E-06 | -0.03(0.02) | 8.44E-02 | 0.03(0.01) | 3.10E-02 |

a-a=amino acid, ch=carbohydrate, c&v=cofactor &vitamin, l=lipid, n=nucleotide, p=peptide, x=xenobiotic
